# Depletion of signal recognition particle 72kDa increases radiosensitivity

**DOI:** 10.1080/15384047.2017.1323587

**Published:** 2017-05-11

**Authors:** Remko Prevo, Gaganpreet S. Tiwana, Timothy S. Maughan, Francesca M. Buffa, W. Gillies McKenna, Geoff S. Higgins

**Affiliations:** Cancer Research UK/ MRC Oxford Institute for Radiation Oncology, Gray Laboratories, Department of Oncology, University of Oxford, Oxford, UK

**Keywords:** Apoptosis, clonogenic assay, radiosensitivity, radiotherapy, signal recognition particle, siRNA, SRP72, unfolded protein response

## Abstract

The identification of genetic determinants that underpin tumor radioresistance can help the development of targeted radiosensitizers or aid personalization of radiotherapy treatment. Here we identify signal recognition particle 72kDa (SRP72) as a novel gene involved in radioresistance. Knockdown of SRP72 resulted in significant radiosensitization of HeLa (cervical), PSN-1 (pancreatic), and T24 (bladder), BT-549 (breast) and MCF7 (breast) tumor lines as measured by colony formation assays. SRP72 depletion also resulted in the radiosensitization of normal lung fibroblast cell lines (HFL1 and MRC-5), demonstrating that the effect is not restricted to tumor cells. Increased radiosensitivity was not due to impaired DNA damage signaling or repair as assessed by γ-H2AX foci formation. Instead SRP72 depletion was associated with elevated levels of apoptosis after irradiation, as measured by caspase 3/7 activity, PARP-cleavage and Annexin-V staining, and with an induction of the unfolded protein response. Together, our results show that SRP72 is a novel gene involved in radioresistance.

## Introduction

Radiotherapy is a critical component of cancer treatment, with more than 50% of cancer patients receiving this treatment in high-income countries.^1^ However, because the maximum dose that can be safely delivered to the tumor is limited by the dose tolerance of the normal surrounding tissue,^2^ the amount of radiation given to the patient is often not enough to sterilize the irradiated tumor. If tumor cells could be selectively made more sensitive to radiation, this could improve the effectiveness of radiotherapy. Furthermore, identifying the genetic determinants of tumor radioresistance may enable identification of patients with radioresistant tumors based on molecular profiling. In an effort to identify novel determinants of tumor radiosensitivity, we recently screened a ‘kinome’ siRNA library, and identified several potential targets.^3^ In this paper, we describe how one of these targets, signal recognition particle 72kDa (SRP72), modulates radiosensitivity. SRP72 was chosen for further investigation because it had not been previously associated with radiosensitivity and Oncomine data suggested an elevated expression in tumor samples.^4^ This differential expression between tumor and normal tissues raised the possibility that disruption of SRP72 might cause tumor specific effects and therefore be an effective therapeutic strategy.

SRP72 is part of a ribonucleoprotein complex, known as the signal recognition particle (SRP), which targets secretory proteins to the rough endoplasmic reticulum, ahead of being integrated as trans-membrane or secreted proteins.^5^ The importance of this is to help secretory proteins travel across the hydrophobic lipid bi-layers, preventing the proteins from obtaining unfavorable structural conformations.^5^ The SRP binds to the hydrophobic residues of the nascent secretory protein,^6,7^ which prevents further elongation of the polypeptide. The SRP then targets the ribosome bound nascent chain to the rough endoplasmic reticulum by binding to the SRP receptor.^8-11^ When the SRP complex disassociates from the ER, elongation resumes and translocation commences. SRP72 is the least characterized subunit of the SRP ribonucleoprotein complex,^12-14^ and its role in tumor radioresistance is unknown. The aim of this study is to validate SRP72 as a modulator of tumor radiosensitivity.

## Results

### SRP72 depletion increases radiosensitivity

**Figure 1.**
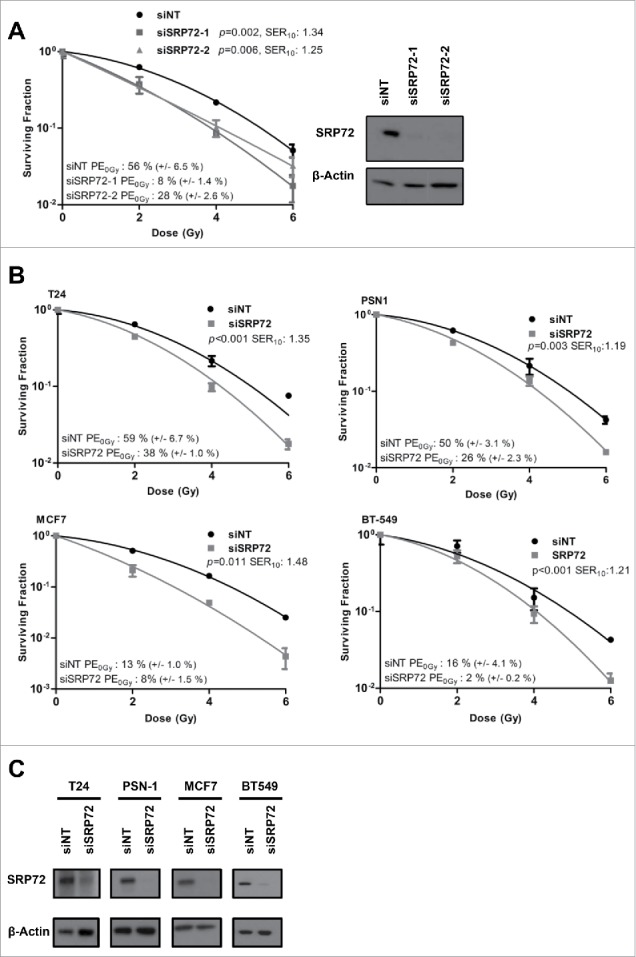
SRP72 knockdown causes tumor cell radiosensitization. (A) Colony forming assay of HeLa cells irradiated following transfection with non-targeting (siNT), siSRP72–1 and siSRP72–2 siRNA. Knockdown was confirmed by immunoblotting (inset). (B) Colony forming assay of T24, PSN-1, MCF7 and BT-549 cells, irradiated following transfection with siNT and siSRP72–1. (C) Knockdown of siRNA transfections from (B) confirmed by immunoblotting. Colony forming assay: representative of n = 3 experiments, data show the mean +/− SD from triplicate wells, *p*-values generated by factorial 2-way ANOVA and the sensitization enhancement ratio at 10% surviving fraction (SER_10_) are indicated.

Following the identification of SRP72 in the siRNA library screen,^3^ we confirmed that this finding was not due to off-target effects of the library siRNA, by using 2 separate siRNA from a vendor different from the one used in the screen. Knockdown of SRP72 significantly radiosensitized HeLa cells, producing SER_10_ values of 1.34 and 1.25 for siSRP72–1 and siSRP72–2, respectively (Fig. 1A). Knockdown of gene products were confirmed by immunoblotting. The 0 Gy plating efficiencies (PE) for SRP72 knockdown cells were lower than the non-targeting control siRNA (siNT), suggesting that depletion of SRP72 also reduces cell viability in the absence of radiation.

To demonstrate that the observed radiosensitivity was not exclusive to HeLa cells, colony formation assays were conducted in cell lines derived from tumor types where radiotherapy plays an important role in routine clinical practice. The T24 (bladder carcinoma), PSN-1 (pancreatic adenocarcinoma), MCF7 and BT-549 (2 breast ductal carcinoma) cell lines were all radiosensitized following depletion of SRP72 using siSRP72–1. Efficient knockdown was confirmed for all cell lines (Fig. 1C). Reduced plating efficiency was again observed following SRP72 suppression (Fig. 1B). In the head and neck squamous cell carcinoma cell line SQ20B, SRP72 knockdown was completely lethal (Plating efficiency was 0% for SRP72 siRNA versus 31% for siNT).

**Figure 2.**
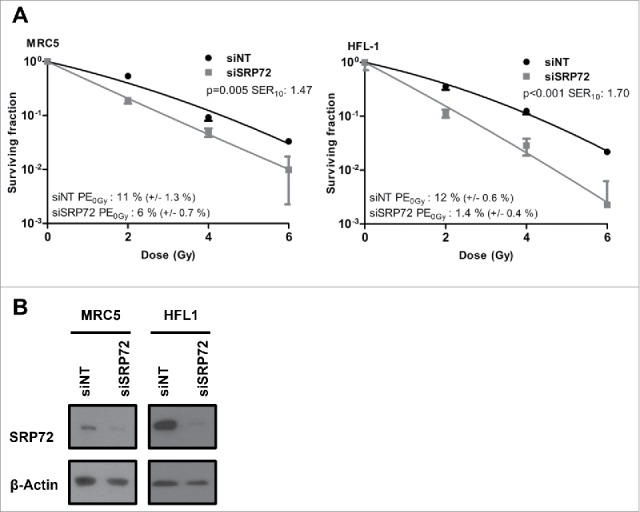
SRP72 knockdown radiosensitizes normal tissue cell lines. (A) Colony forming assay of MRC-5 and HFL1 cells irradiated following transfection with siNT and siSRP72–1. (B) Confirmation of knockdown by immunoblotting. Colony forming assay: representative of n = 3 experiments, data show the mean +/− SD from triplicate wells, *p*-values generated by factorial 2-way ANOVA and the sensitization enhancement ratio at 10% surviving fraction (SER_10_) are indicated.

To gauge whether SRP72 could be a potential therapeutic target, we sought to determine whether the radiosensitization was tumor specific. However, the normal lung fibroblast cell lines, MRC-5 and HFL1 were both significantly radiosensitized following the knockdown of SRP72 (Fig. 2A, B), suggesting that targeting SRP72 in cancer patients is unlikely to improve the therapeutic window.

### SRP72 depletion potentiates apoptosis

**Figure 3.**
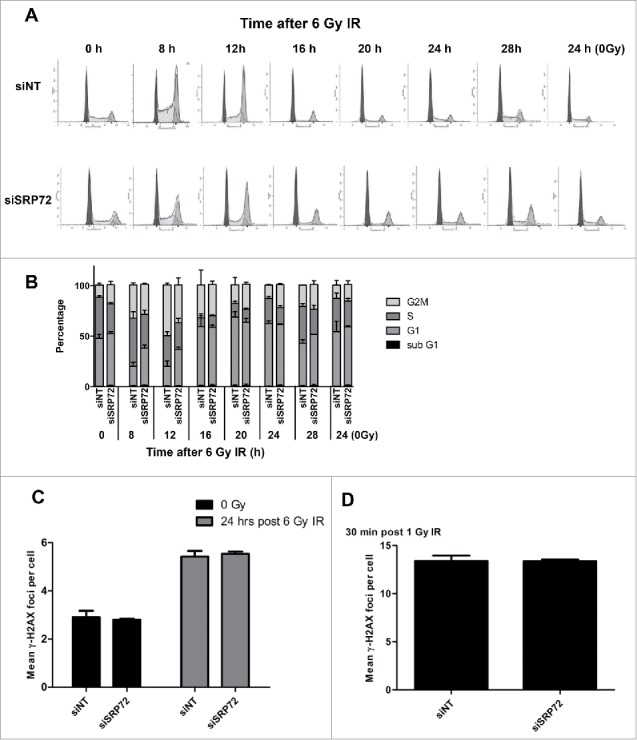
Cell cycle profile and DNA-damage foci formation following SRP72 knockdown and irradiation. (A) Cell cycle profile of HeLa cells irradiated 72 hours after transfection with siSRP72–1. Cells were fixed at different time points post-irradiation and stained with propidium iodide followed by flow cytometry and curve fitting with ModFit software (dark gray: G1, white: S; light gray: G2/M); representative of n = 3 experiments. (B) Quantification of the cell cycle profiles and sub-G1 population from 3 individual experiments. (C) DNA damage foci at 24 hours after IR. HeLa cells were irradiated at 6 Gy 72 hours after transfection with siSRP72–1, fixed 24 hours later and probed for γ-H2AX. Foci were imaged and quantified using the IN Cell analyzer, counting at least 500 cells per well. (D) DNA damage foci at 30 min after IR. HeLa cells were irradiated at 1 Gy 72 hours after siRNA transfection with siSRP72–1 and fixed 30 min later and treated as described in (C). Representative experiment of n = 3 is shown. Foci data are the mean +/− SD from triplicate wells. Unpaired 2-sided students t-tests comparing siNT to siSRP72, **p < 0.01. Radiation doses of 1 Gy and 6 Gy were used for the 30 min and 24h foci time points, respectively, to ensure optimal foci quantification.

Next we sought to identify the mechanism by which SRP72-depleted cells died following radiation. HeLa cells were used for these mechanistic experiments because of the efficient and reproducible SRP72 knockdown that was achieved in this cell line. In the first instance we investigated whether there were any differences in radiation-induced cell cycle arrest following SRP72 depletion. Knockdown of SRP72 in HeLa cells resulted in a reduced radiation-induced G2 arrest compared with siNT (Fig. 3A). Although the reduction was not statistically significant across separate experiments (Fig. 3B), this observation does suggest an effect on cell cycle checkpoints. We subsequently looked into DNA repair following radiation. DNA damage foci quantification showed that cells with SRP72 knockdown showed no significant difference in γ-H2AX foci at 30 min and 24 hours after irradiation when compared with siNT (Fig. 3C and D). This suggests that the observed radiosensitization is not the result of increased radiation-induced DNA damage or reduced DNA repair.

**Figure 4.**
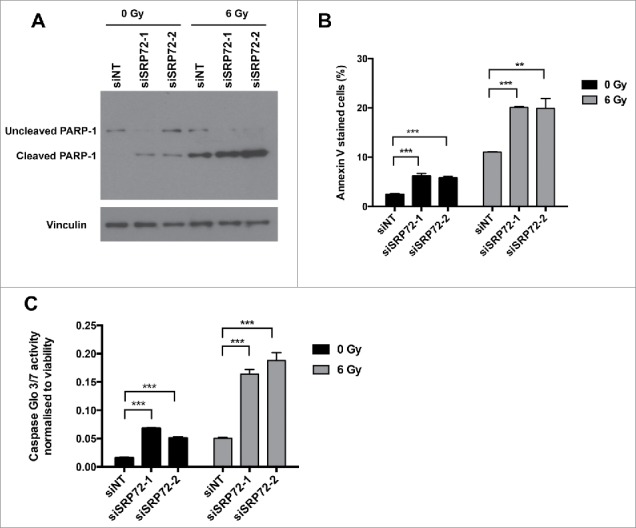
SRP72 knockdown potentiates apoptosis following IR. HeLa cells were irradiated with 6 Gy IR 72 hours after siRNA transfection with siNT and siSRP72–1 and siSRP72–2. (A) Immunoblotting with cleaved PARP-1 antibody on cell lysates obtained 72 hours post irradiation, representative experiment of n = 3. (B) Annexin-V/PI staining performed 72 hours post irradiation and samples analyzed by flow cytometry, representative experiment of n = 3 is shown, data represented as mean +/− SD from triplicate wells. Unpaired 2-sided students t-tests comparing siNT to siSRP72 knockdown, **p < 0.01, ***p < 0.001. (C) Caspase 3/7 activity measured 72 hours post irradiation and normalized to cell viability readout. Representative experiment of n = 3 is shown, data show the mean +/− SD from triplicate wells. Unpaired 2-sided students t-tests comparing siNT to siSRP72 knockdown, ***p < 0.001.

Subsequently, we investigated whether elevated apoptosis could explain the increased cell death. Analysis of the sub-G1 population in the cell cycle profiles shown earlier did not identify a significant increase in apoptosis (Fig. 3B). However, these analyses were done at early time points, and we therefore decided to assess apoptosis 3 d after irradiation. Levels of apoptosis were investigated following knockdown and irradiation of SRP72-depleted cells. Poly (ADP-ribose) polymerase (PARP) cleavage when measured by immunoblotting following IR was strongly increased comparing siNT to siSRP72 (Fig. 4A). The elevation in apoptosis was further confirmed by Annexin-V PI staining and Caspase activity assays (Fig. 4B, C). Knockdown of SRP72 also caused an increase in Caspase 3/7 activity and Annexin-V staining in the absence of radiation, mirroring the reduced plating efficiencies seen in the clonogenic assays. These assays were performed using both SRP72 siRNAs, siSRP72–1 and siSRP72–2, further confirming the absence of off-target effects.

### SRP72 depletion does not affect protein synthesis and induces the unfolded protein response

**Figure 5.**
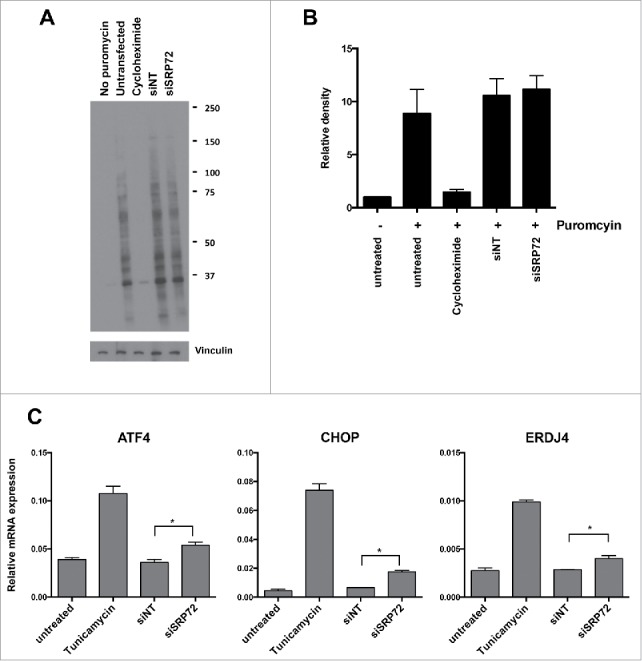
SRP72 depletion induces the unfolded protein response. (A) SRP72 depletion does not affect protein synthesis. Four days after transfection with siSRP72–1, HeLa cells were labeled with puromycin in the presence or absence of the translation inhibitor cycloheximide. Cell lysates were analyzed by Western blotting with anti-puromycin antibody. Representative experiment of n = 3 is shown. (B) Quantification of the Western blots from 3 experiments by densitometric scanning and normalizing to the vinculin loading controls and no-puromycin lanes. Data show the mean +/− SD. (C) Expression levels of 3 markers for the unfolded protein response. Four days after transfection with siSRP72–1 or 24 hours after treatment with tunicamycin, mRNA levels of ATF4, CHOP, ERDJ4 were analyzed by qRT-PCR. Representative experiment of n = 3 is shown, data show the mean +/− SD from duplicate wells. Unpaired 2-sided students t-tests comparing siNT to siSRP72 knockdown, *p < 0.05.

As SRP72 is involved in the targeting of polypeptides across the endoplasmatic reticulum, we investigated whether its depletion affected protein synthesis using the non-radioactive SUnSET assay.^15^ This method measures the incorporation of puromycin, a structural analog of aminoacyl tRNA, into nascent polypeptides, which can subsequently be detected with a puromycin antibody. Using this assay, we could not detect any apparent decrease in protein synthesis in SRP72 siRNA transfected cells (Fig. 5A, B). Effective protein synthesis inhibition was demonstrated after incubation cycloheximide, which interferes with the translocation step in protein synthesis, thus validating the assay.

As it is conceivable that disruption of the signal recognition complex leads to accumulation of misfolded proteins triggering the unfolded protein response, we investigated whether this was the case following SRP72 depletion. We measured the induction of 3 genes known to be induced during the unfolded protein response, ATF4, CHOP, and ERDJ4^16,17^ and used the N-glycosylation inhibitor tunicamycin, a potent ER stress inducer,^17^ as positive control. Indeed we found that the mRNA levels of all 3 genes were increased following siSRP72 transfection (Fig. 5C), confirming that SRP72 depletion induces the unfolded protein response.

## Discussion

In this paper we show for the first time that a component of the signal recognition particle, SRP72, modulates radiosensitivity. The decreased radiosurvival in SRP72-depleted cells was associated with a delayed G2/M arrest but was not caused by an inhibition in DNA damage signaling or repair. Instead we found that the radiosensitivity caused by SRP72 knockdown was associated with an elevation in apoptosis. Increased apoptosis was also found in SRP72-depleted cells in the absence of irradiation. In one cell line, the head and neck cancer line, SQ20B, depletion of SRP72 was completely lethal, suggesting that the degree of SRP72 dependency is cell line specific. The decreased survival in clonogenic survival assays suggests that apoptosis is synergistically increased in the context of irradiation. SRP72 depletion did not affect protein synthesis but it did induce the unfolded protein response, which is likely to be a major cause of the increased levels of apoptosis in these cells.

Although we did not specifically investigate the other components of the signal recognition particle, analysis of our siRNA screen data suggested that depletion of 2 other components, SRP54 and SRPRB, did not significantly increase radiosensitivity (ref.18 and unpublished observations).

There is minimal literature regarding SRP72 and its involvement in cancer progression or radioresistance. A study exploring the differences between how death receptor 4 and 5 (DR4 & DR5) are regulated in response to TRAIL mediated apoptosis identified SRP72 as a regulator of this response.^19^ The authors found that depleting SRP72 in HCT15 cells conferred resistance against DR4 dependent apoptosis.^19^ In contrast to our results, they did not see a reduction in cell viability or growth arrest with SRP72 knockdown.

Depletion of SRP72 caused radiosensitization in tumor lines derived from several histological sites suggesting that it is not restricted to tumor type. However, as non-cancerous lung fibroblast cells were also radiosensitized by SRP72 depletion, drug inhibition of SRP72 is unlikely to be a useful therapeutic strategy, as it is not anticipated to improve the therapeutic window for radiotherapy treatment. Nevertheless, expression of SRP72 may be associated with radioresistance and we are currently assessing whether this gene will be included in an ‘intrinsic radiosensitivity’ signature to predict patients' response to radiotherapy.

## Materials and methods

### Cell culture

HeLa, BT-549, PSN-1, T24, MRC-5, HFL1 and MCF7 cells were purchased from American Type Culture Collection (ATCC). SQ20B cells were supplied by Dr. Ralph Weichselbaum (University of Chicago). HeLa, BT-549, PSN-1 and MCF7 were cultured in Dulbecco's modified Eagle's medium (DMEM) and T24 in RPMI. MRC-5 cells were cultured in minimum essential medium (MEM). HFL1 cells were cultured in DMEM/F-12. Media (Sigma) were supplemented with 10% Fetal Bovine Serum (FBS). Cells were regularly tested for mycoplasma using the MycoAlert kit (Lonza). Cell lines grown beyond 4 months after purchase were authenticated by the DNA Diagnostics Center by short tandem repeat (STR) profiling.

### siRNA transfections

Cells were transfected in 6-well plates with siRNA using INTERFERin-HTS (Polyplus) transfection reagent in a reverse transfection procedure. Ambion Silencer Select siRNA (20 nMol/L; Life Technologies) was used for all assays. The sense strand sequences for SRP72 were as follows: SRP72 (1): GGACAAGUGUUAUACCGUU and SRP72 (2): GGCAAUUAGUGACCUACAA. Silencer Select Negative Control No. 1 siRNA was used as negative control. The volume of INTERFERin-HTS ranged from 1 µl to 2 µl per well and the seeding density from 150,000 to 200,000 cells per well, depending on the cell line. Cells were re-plated for colony formation assays, knockdown confirmation, and other downstream assays 72 hours after transfection.

### Colony formation assays

Single cell suspensions of siRNA transfected cells were plated in 6-well plates and left 4 hours at 37°C (5% CO_2_) to adhere. For unirradiated plates, 200 cells were plated per well (1000 cells in the case for MRC-5 and HFL1 cells) and this number was doubled for each 2 Gy of increased radiation dose. Plates were irradiated at 2, 4 and 6 Gy using a caesium-137 irradiator, Gamma Service: GSR D1; dose rate 1.938 Gy min^−^1. Colonies were grown for 10–14 d and stained with crystal violet.

Treatment effects on dose survival curves were calculated as follows:

a) Survival data was fitted using non-linear regression with the linear quadratic equation: S = exp - (αD + βD^2^), *S* denotes survival probability, D (Gy) is radiation dose and α (Gy^−1^) and β (Gy^−2^) are parameter constants. An indicator variable was introduced to specify targeting and non-targeting condition as described previously.^20^ The sensitization enhancement ratio at 10% surviving fraction (SER_10_) was calculated: SER10: D*_untreated_* / D*_treated_*, where D*_untreated_* and D*_treated_* yield 10% survival as calculated using α (Gy^−1^) and β (Gy^−2^) parameters.
b) A factorial 2-way ANOVA was performed with survival as the dependent variable and dose levels (2, 4 and 6 Gy) and treatment (siRNA) as the 2 factors; interaction between dose and treatment was estimated.

### Immunoblotting

Protein lysates were prepared using RIPA lysis buffer (Thermo Scientific) and proteins were separated using SDS-PAGE electrophoresis followed by immunoblotting. Bound antibodies against SRP72 (Epitomics, #T1841) and cleaved PARP-1 (New England Biolabs, #9541) were detected by developing film exposed to nitrocellulose membrane incubated with chemiluminescence reagent (SuperSignal, Millipore).

### Immunofluorescence

siRNA-transfected cells were re-plated into 96-well plates and left to adhere for 4 hours at 37°C before irradiation. Plates were then fixed at different time points using 3% formaldehyde. Cells were probed for γ-H2AX (Upstate/Millipore, #05–636) antibody as described previously,^21^ and foci were imaged and quantified using an IN Cell Analyzer (GE).

### Flow cytometry

siRNA-transfected HeLa cells were re-plated into 6-well plates at a concentration of 1 × 10^5^ cells/well. Plates were left to adhere for 4 hours and irradiated at 6 Gy. For cell cycle analysis, cells were fixed at different time points using ice cold 70% ethanol and after a PBS wash stained in Propidium iodide (PI) staining solution (50 μg/ml PI, 200 μg/ml RNase). Stained cells were run on a BD FACScan flow cytometer and plots analyzed using ModFit software. For Annexin-V/PI staining, cells were stained using the Annexin-V-FLUOS staining kit (Roche) as per manufacturer's instructions. Samples were run on a FACSCalibur and analyzed by CellQuest (Becton Dickinson).

### Caspase 3/7 activity assay

Cells were plated into 96-well plates and left to adhere for 4 hours at 37°C before irradiation at 6 Gy and incubated for another 72 hours. Prior to caspase activity measurement, cells were first incubated with 50µl 10 µg/ml resazurin (Sigma) in medium for 1 hour before fluorescence measurement at 560Ex/590Em on a POLARstar Omega plate reader to assess cell number. Subsequently, 50 µl of Caspase-Glo 3/7 assay (Promega) solution was added, plates were left for 1 hour at room temperature and luminescence was read using a POLARstar Omega plate reader. Luminescence values were normalized to cell number fluorescence values to account for cell number differences between wells.

### Measurement of protein synthesis

Cells were incubated with 10 µg/ml puromycin for 30 min at 37°C, followed by a further 60 min at 37°C after washout of the puromycin. Cycloheximide (20 µM) was added 20 min before puromycin addition as a positive control for inhibition of protein synthesis. Protein lysates were prepared as described above and incorporated puromycin was detected by Western blotting with monoclonal anti-puromycin (Millipore, #MABE343).

### qRT-PCR for unfolded protein response

Four days after siRNA transfection or 24 hour after treatment with 1 µg/ml tunicamycin, RNA was extracted using the RNeasy kit (Qiagen) followed by cDNA synthesis using the qPCRBIO cDNA synthesis kit (PCR Biosystems). PCR was performed with the Brilliant II SYBR green kit (Agilent Technologies) using the following primers. GAPDH-F: CCGCATCTTCTTTTGCGTCGC, GAPDH-R: AAATGAGCCCCAGCCTTCTCCATG. ATF4 F: TGACCTGGAAACCATGCCAG, ATF4 R: AATGATCTGGAGTGGAGGAC, CHOP F: GGAGCATCAGTCCCCCACTT, CHOP R: TGTGGGATTGAGGGTCACATC, ERDJ4 F: AAAATAAGAGCCCGGATGCT, ERDJ4 R: CGCTTCTTGGATCCAGTGTT. Ct values were converted into relative copy number and normalized to the GAPDH control.

## References

[1] Atun R, Jaffray DA, Barton MB, Bray F, Baumann M, Vikram B, Hanna TP, Knaul FM, Lievens Y, Lui TY, et al. Expanding global access to radiotherapy. Lancet Oncol 2015; 16:1153-86; PMID:26419354; https://doi.org/10.1016/S1470-2045(15)00222-3 26419354

[2] Emami B, Lyman J, Brown A, Coia L, Goitein M, Munzenrider JE, Shank B, Solin LJ, Wesson M Tolerance of normal tissue to therapeutic irradiation. Int J Radiat Oncol Biol Phys 1991; 21:109-22; PMID:2032882; https://doi.org/10.1016/0360-3016(91)90171-Y 2032882

[3] Tiwana GS, Prevo R, Buffa FM, Yu S, Ebner DV, Howarth A, Folkes LK, Budwal B, Chu KY, Durrant L, et al. Identification of vitamin B1 metabolism as a tumor-specific radiosensitizing pathway using a high-throughput colony formation screen. Oncotarget 2015; 6(8):5978-89; PMID:25788274; https://doi.org/10.18632/oncotarget.3468 25788274 PMC4467415

[4] Rhodes DR, Kalyana-Sundaram S, Mahavisno V, Varambally R, Yu J, Briggs BB, Barrette TR, Anstet MJ, Kincead-Beal C, Kulkarni P, et al. Oncomine 3.0: genes, pathways, and networks in a collection of 18,000 cancer gene expression profiles. Neoplasia 2007; 9:166-80.PMC1813932; PMID:17356713; https://doi.org/10.1593/neo.07112 17356713 PMC1813932

[5] Lutcke H Signal recognition particle (SRP), a ubiquitous initiator of protein translocation. Euro J Biochem 1995; 228:531-50; PMID:7737147; https://doi.org/10.1111/j.1432-1033.1995.tb20293.x 7737147

[6] Zopf D, Bernstein HD, Johnson AE, Walter P The Methionine-rich domain of the 54 Kd protein subunit of the signal recognition particle contains an Rna-Binding site and can be cross-linked to a signal sequence. Embo J 1990; 9:4511-7; PMID:1702385; https://www.ncbi.nlm.nih.gov/pmc/articles/PMC552245/pdf/emboj00240-0298.pdf 1702385 10.1002/j.1460-2075.1990.tb07902.x PMC552245

[7] Lutcke H, High S, Romisch K, Ashford AJ, Dobberstein B The Methionine-Rich Domain of the 54 Kda Subunit of Signal Recognition Particle Is Sufficient for the Interaction with Signal Sequences. Embo J 1992; 11:1543-51; PMID:1314169; https://www.ncbi.nlm.nih.gov/pmc/articles/PMC556603/pdf/emboj00089-0314.pdf 1314169 10.1002/j.1460-2075.1992.tb05199.x PMC556603

[8] Gilmore R, Walter P, Blobel G Protein translocation across the endoplasmic reticulum. II. Isolation and characterization of the signal recognition particle receptor. J Cell Biol 1982; 95:470-7.2112977; PMID:6292236; https://doi.org/10.1083/jcb.95.2.470 6292236 PMC2112977

[9] Fulga TA, Sinning I, Dobberstein B, Pool MR SRbeta coordinates signal sequence release from SRP with ribosome binding to the translocon. Embo J 2001; 20:2338-47.125438; PMID:11331598; https://doi.org/10.1093/emboj/20.9.2338 11331598 PMC125438

[10] Jiang Y, Cheng ZL, Mandon EC, Gilmore R An interaction between the SRP receptor and the translocon is critical during cotranslational protein translocation. J Cell Biol 2008; 180:1149-61; PMID:18347066; https://doi.org/10.1083/jcb.200707196 18347066 PMC2290843

[11] Rapiejko PJ, Gilmore R Empty site forms of the SRP54 and SR alpha GTPases mediate targeting of ribosome-nascent chain complexes to the endoplasmic reticulum. Cell 1997; 89:703-13; PMID:9182758; https://doi.org/10.1016/S0092-8674(00)80253-6 9182758

[12] Walter P, Blobel G Purification of a membrane-associated protein complex required for protein translocation across the endoplasmic-reticulum. P Natl Acad Sci-Biol 1980; 77:7112-6; PMID:6938958; https://doi.org/10.1073/pnas.77.12.7112 6938958 PMC350451

[13] Walter P, Ibrahimi I, Blobel G Translocation of proteins across the endoplasmic-reticulum .1. signal recognition protein (Srp) binds to invitro-assembled polysomes synthesizing secretory protein. J Cell Biol 1981; 91:545-50; PMID:7309795; https://doi.org/10.1083/jcb.91.2.545 7309795 PMC2111968

[14] Walter P, Blobel G Signal recognition particle contains a 7S RNA essential for protein translocation across the endoplasmic reticulum. Nature 1982; 299:691-8; PMID:6181418; https://doi.org/10.1038/299691a0 6181418

[15] Schmidt EK, Clavarino G, Ceppi M, Pierre P SUnSET, a nonradioactive method to monitor protein synthesis. Nat Methods 2009; 6:275-7; PMID:19305406; https://doi.org/10.1038/nmeth.1314 19305406

[16] Harding HP, Novoa I, Zhang Y, Zeng H, Wek R, Schapira M, Ron D Regulated translation initiation controls stress-induced gene expression in mammalian cells. Mol Cell 2000; 6:1099-108; PMID:11106749; https://doi.org/10.1016/S1097-2765(00)00108-8 11106749

[17] Samali A, Fitzgerald U, Deegan S, Gupta S Methods for monitoring endoplasmic reticulum stress and the unfolded protein response. Int J Cell Biol 2010; 2010:830307.PMC2821749; PMID:20169136; https://doi.org/10.1155/2010/830307 20169136 PMC2821749

[18] Tiwana GS, Prevo R, Buffa FM, Yu S, Ebner DV, Howarth A, Folkes LK, Budwal B, Chu KY, Durrant L, et al. Identification of vitamin B1 metabolism as a tumor-specific radiosensitizing pathway using a high-throughput colony formation screen. Oncotarget 2015; 6:5978-89.PMC4467415; PMID:25788274; https://doi.org/10.18632/oncotarget.3468 25788274 PMC4467415

[19] Ren YG, Wagner KW, Knee DA, Aza-Blanc P, Nasoff M, Deveraux QL Differential regulation of the TRAIL death receptors DR4 and DR5 by the signal recognition particle. Mol Biol Cell 2004; 15:5064-74; PMID:15356269; https://doi.org/10.1091/mbc.E04-03-0184 15356269 PMC524775

[20] Chalmers AJ, Bentzen SM, Buffa FM A general framework for quantifying the effects of DNA repair inhibitors on radiation sensitivity as a function of dose. Theor Biol Med Model 2007; 4:4; PMID:17254360; https://doi.org/10.1186/1742-4682-4-25 17640390 PMC1950494

[21] Higgins GS, Prevo R, Lee YF, Helleday T, Muschel RJ, Taylor S, Yoshimura M, Hickson ID, Bernhard EJ, McKenna WG A small interfering RNA screen of genes involved in DNA repair identifies tumor-specific radiosensitization by POLQ knockdown. Cancer Res 2010; 70:2984-93.PMC2848966; PMID:20233878; https://doi.org/10.1158/0008-5472.CAN-09-4040 20233878 PMC2848966

